# Identification of Significant Pathways Induced by PAX5 Haploinsufficiency Based on Protein-Protein Interaction Networks and Cluster Analysis in Raji Cell Line

**DOI:** 10.1155/2017/5326370

**Published:** 2017-02-21

**Authors:** Jia Gu, TongJuan Li, Lei Zhao, Xue Liang, Xing Fu, Jue Wang, Zhen Shang, Wei Huang, Jianfeng Zhou

**Affiliations:** Department of Hematology, Tongji Hospital, Tongji Medical College, Huazhong University of Science and Technology, Wuhan, Hubei, China

## Abstract

PAX5 encodes a transcription factor essential for B-cell differentiation, and PAX5 haploinsufficiency is involved in tumorigenesis. There were few studies on how PAX5 haploinsufficiency regulated genes expression to promote tumorigenesis. In this study, we constructed the cell model of PAX5 haploinsufficiency using gene editing technology in Raji cells, detected differentially expressed genes in PAX5 haploinsufficiency Raji cells, and used protein-protein interaction networks and cluster analysis to comprehensively investigate the cellular pathways involved in PAX5 haploinsufficiency. The clusters of gene transcription, inflammatory and immune response, and cancer pathways were identified as three important pathways associated with PAX5 haploinsufficiency in Raji cells. These changes hinted that the mechanism of PAX5 haploinsufficiency promoting tumorigenesis may be related to genomic instability, immune tolerance, and tumor pathways.

## 1. Introduction

The paired box domain gene 5 (PAX5) encodes a paired box domain (PBD) transcription factor essential for B-cell differentiation that activates crucial genes for B-cell lineage differentiation and represses genes important for commitment in other hematopoietic lineages [1, 2]. Mutation of PAX5 participates in B-cell tumorigenesis [3, 4]. Conditional PAX5 deletion in mice allowed mature B-cells from peripheral lymphoid organs to dedifferentiate in vivo back to early uncommitted progenitors in the bone marrow. Mice lacking PAX5 in mature B-cells also developed aggressive lymphomas [5]. PAX5 haploinsufficiency synergized with STAT5 activation to initiate acute lymphoblastic leukemia (ALL) and the probability of tumor formation was 100% [6]. PAX5 haploinsufficiency cooperated with BCR-ABL1 to induce acute lymphoblastic leukemia [1].

PAX5 has been reported as being frequently altered in both childhood [2] and adult [7, 8] B-ALL. PAX5 mutation was also reported in both Hodgkin lymphoma [9] and non-Hodgkin lymphoma [10, 11]. PAX5 genomic deletions were predicted to result in PAX5 haploinsufficiency or expression of PAX5 isoforms with impaired DNA binding [7, 12, 13], which resulted in PAX5 haploinsufficiency. So, PAX5 haploinsufficiency plays an important role in lymphocytic neoplasm.

We do not know how PAX5 haploinsufficiency regulates genes expression to promote tumorigenesis, though previous studies showed heterozygous mice (PAX5+/−) had higher penetrance of B-ALL than wild-type mice (PAX5+/+) [14, 15]. The transcription factor PAX5 is pivotal for B-cell commitment in mice. It represses lineage-inappropriate gene expression while concurrently activating the B-cell gene expression program. Clare Pridans performed global gene expression screen of wild-type (PAX5+/+) and PAX5-deletion (PAX5−/−) pro-B-cells in an attempt to identify the crucial PAX5 targets in early B-lymphopoiesis. He identified 109 PAX5 targets comprising 61% activated and 39% repressed genes [16]. A key function of PAX5 is to activate secondary transcription factors that further reinforce the B-cell program [16]. The allele-specific regulation of PAX5 is random, reversible, and independent of parental origin and correlates with synchronous replication during B-cell development. The allele-specific regulation of PAX5 may be a common mechanism causing the haploinsufficiency and frequent association of other PAX genes with human disease [17]. Cell model of PAX5 haploinsufficiency is more similar to the human disease state, and PAX5 haploinsufficiency (PAX5+/−) may have different effect on target genes compared with PAX5−/− at cell level, but there were few studies on PAX5 haploinsufficiency on genes expression at the cellular level.

In our study, we constructed a new cell model of PAX5+/− using gene editing technology which knocked out one PAX5 allele in Raji cell line (lymphoblastoid cell line derived from Burkitt lymphoma). We analyzed the gene expression profile in PAX5+/− Raji cells and their mother wild-type cells. We constructed the protein-protein interaction (PPI) network of the differentially expressed genes and screened out the most significant subnetwork. In addition, the enriched functions and pathways of DEGs were used to identify significant pathways involved in PAX5 haploinsufficiency Raji cells. This study was very valuable for our understanding of how PAX5 haploinsufficiency regulated genes expression to promote tumorigenesis.

## 2. Materials and Methods

### 2.1. Cell Culture

The lymphoblastoid cell line Raji was purchased from China Center for Type Culture Collection (CCTCC, Wuhan, China) and HEK293T was purchased from the American Type Culture Collection (ATCC, United States). Raji cells were cultured in RPMI1640 medium containing 5~10% fetal calf serum (FCS, Invitrogen, United States), and HEK293T cells were cultured in DMEM supplemented with 10% dialyzed fetal bovine serum.

### 2.2. Construction and Validation of Customized CRISPR/CAS9 Expression Vectors

The vector pSpCas9(BB)-2A-GFP (PX458) (Plasmid #48138) was purchased from Addgene (Massachusetts, USA). The oligo-DNA targeting the PAX5 exon5 locus was designed on the MIT online software ZhangFeng lab: http://crispr.mit.edu/. We selected three high scored sequences and designed their respective complement chains with restriction site; each single strand oligo-DNA chain was synthesized in Invitrogen company as follows:

  PAX5 gRNA-F1: cacc  GACAAAAGTACAGCAGCCAC
  PAX5 gRNA-R1: aaac  GTGGCTGCTGTACTTTTGTC
  PAX5 gRNA-F2: cacc  AACCAACCAGTCCCAGCTTC
  PAX5 gRNA-R2: aaac  GAAGCTGGGACTGGTTGGTT
  PAX5 gRNA-F3: cacc  ACCAACCAGTCCCAGCTTCC
  PAX5 gRNA-R3: aaac  GGAAGCTGGGACTGGTTGGT

Next, we annealed the two complement chains to form dsDNA using Precut sgRNA Cloning kit and pSD-gRNA Plasmid construction Kit (Biomics Biotechnologies Co., Ltd., Jiangsu, China) according to the instruction. This was followed by BbsI digestion and ligation with T4 ligase to construct Cas9/sgRNA plasmids targeting PAX5. The Cas9/sgRNA plasmids were amplified, purified with EndoFree Plasmid Maxi Kit (QIAGEN, Germany), and validated by sequencing. The Cas9/sgRNA plasmids were electrotransfected into HEK293T cells. The T7 Endonuclease I assay was applied to measure the NHEJ-mediated mutations efficiency in the endogenous PAX5 gene. The most efficient Cas9/sgRNA plasmid (gRNA-F1/R1) was chosen for subsequent research.

### 2.3. Construction and Identification of PAX5+/− Raji Cell Clone

5 × 10^5^~2 × 10^6^ Raji cells in good condition were collected and suspended with matched solution supplemented with 5 *μ*g CRISPR/CAS9 plasmid. Electrotransfection was performed with optimized program on LONZA 4D Nucleofector System (Lonza, Switzerland). Cells were cultured for 48 h and then sorted with Beckman MoFlo XDP (Beckman Coulter, Inc., USA), aiming to select cells with high GFP expression. The sorted cells were seeded in 96-well plates in the manner of single cell. Two or three weeks later, cells were collected for identification. Genomic DNA was isolated using the DNA Isolation Kit (BioTeke Corporation, Beijing, China) according to the manufacturer's instructions. PCR was used to amplify the PAX5 gene for mutation analysis. The PCR primers, synthesized by Sangon Biotech (Shanghai, China), were as follows: forward primer CTTCAGAAGAGGCACTTGAAGC and reverse primer TTACCAGGTTCAGCCCTTGG. The PCR product was reclaimed for sequence determination. The sequencing results were compared with the published PAX5 gene sequence to determine the presence of pax5+/− variants.

### 2.4. Western Blot Analysis for PAX5

Cells were lysed with RIPA lysis buffer (Beyotime, China) supplemented with a protease inhibitor cocktail (Roche, Switzerland). The bicinchoninic acid protein assay (Thermo Scientific, USA) was used to measure protein concentration. 40 *μ*g of total lysate was subjected to SDS-PAGE and then transferred to nitrocellulose membranes (Bio-Rad Laboratories, USA). The membranes were incubated with the antibodies against PAX5 and GAPDH purchased from Abcam Biotechnology (Abcam, CA, USA), and then they were blotted with corresponding HRP-linked secondary antibodies. The proteins were detected using an enhanced ECL system (Pierce, USA). Quantification was performed with ImageJ (https://imagej.nih.gov/) and each sample's ratio relative to the loading control GAPDH was calculated.

### 2.5. Quantitative RT-PCR Analysis

Total RNA was extracted with TRIzol (Invitrogen, USA) from the wide-type and mutational cells. A NanoDrop microvolume spectrophotometer (Thermo Fisher) was used to quantify the RNA and RT-PCR was performed with 2 *μ*g of total RNA and oligo-dT, and 1 *μ*L of cDNA was used as quantitative RT-PCR template in 10 *μ*L PCR mix (GeneCopoeia, Guangzhou, China) with 1 *μ*L of primer and 8 *μ*L ddH2O (20 *μ*L reaction volume). Quantitative RT-PCR was performed using a CFX96 Touch™ Real-Time PCR Detection System (Bio-Rad) according to the instructed thermocycler program for each locus. PCR primer sequences, synthesized by BGI, Shenzhen, China, are shown as follows: GAPDH: 5′-GAGTCCACTGGCGTCTTCA-3′ (forward), 5′-GGGTGCTAAGCAGTTGGT-3′ (reverse); CD19: 5′-GGCCCGAGGAACCTCTAGT-3′ (forward), 5′-TAAGAAGGGTTTAAGCGGGGA-3′ (reverse); CD79A: 5′-CAAGAACCGAATCATCACAGCC-3′ (forward), 5′-TCTGCCATCGTTTCCTGAACA-3′ (reverse); FCER2: 5′-CCAGGAATTGAACGAGAGGAAC-3′ (forward), 5′-TTGATCCACTTTTCA GGGCAC-3′ (reverse); IGLL1: 5′-ACCCAGCTCACCGTTTTAAGT-3′ (forward), 5′-GGTCACCGT CAAGATTCCCG-3′ (reverse).

### 2.6. Microarray Assay

Total RNA was exacted using TRIzol reagent (Invitrogen, USA) according to the manufacturer's protocol. The experimental samples' RNA integrity number (RIN) was confirmed to be no less than 7.0 to ensure the quality and quantity. Library construction and RNA sequencing were performed at Beijing Genomics Institute (BGI, Shenzhen). The final libraries were quantitated by the Agilent 2100 bioanalyzer instrument (Agilent DNA 1000 Reagents) to ensure the size and purity of the sample and then sequenced using the HiSeq 2000 System (TruSeq SBS KIT-HS V3, Illumina), with read length 50.

After being aligned to the human reference genome (NCBI Build 36.1) using SOAPaligner-v2.21 software (BGI) with high-quality reads, the matched data were aligned with reference sequence on Human RefSeq mRNA (NCBI). We normalized the expression levels for each gene to the reads per kilobase of the target exon per million mapped reads (RPKM) to facilitate the comparison of transcripts between samples. The differentially expressed genes (DEGs) between each pair were identified using the standard of “FDR ≤ 0.001 and the absolute value of log 2 Ratio ≥ 2.”

### 2.7. The Motif Analysis of PAX5 and Identification of PAX5 Target Genes

The motif analysis of PAX5 and identification of PAX5 target genes were accomplished based on TRRUST Database (http://www.grnpedia.org/trrust/result.php?gene=PAX5) and TRED Database (https://cb.utdallas.edu/cgi-bin/TRED/tred.cgi?process=searchTFGeneForm).

### 2.8. Construction of PPI Network and Subnetwork

The online database STRING (Search Tool for the Retrieval of Interacting Genes) offers uniquely wide coverage and ease of access to both laboratorial and predicted interaction information [18]. In our study, the interactions between DEGs were derived based on STRING and the associations with a correlation coefficient > 0.8 were recognized as PPIs. The PPI network was constructed and visualized using Cytoscape software, as formerly described [19]. Cytoscape is an open software project for integrating biomolecular interaction networks with high-throughput expression data and other molecular states into a unified conceptual framework.

### 2.9. Pathway Enrichment Analysis

The DAVID (Database for Annotation, Visualization and Integrated Discovery) contains an integrated biological knowledge base and analytic tools, with the purpose of extracting biological meaning from large gene/protein lists [20]. The KEGG (Kyoto Encyclopedia of Genes and Genomes) is a knowledge base for the analysis of gene functions, connecting genomic information with higher order functional information [21]. In our study, pathway enrichment analysis was administered for the PPI network by DAVID and the significantly enriched pathways were confirmed with a value of *p* < 0.05.

## 3. Results

### 3.1. Construction and Identification of PAX5+/− Raji Cells

**Figure 1:**
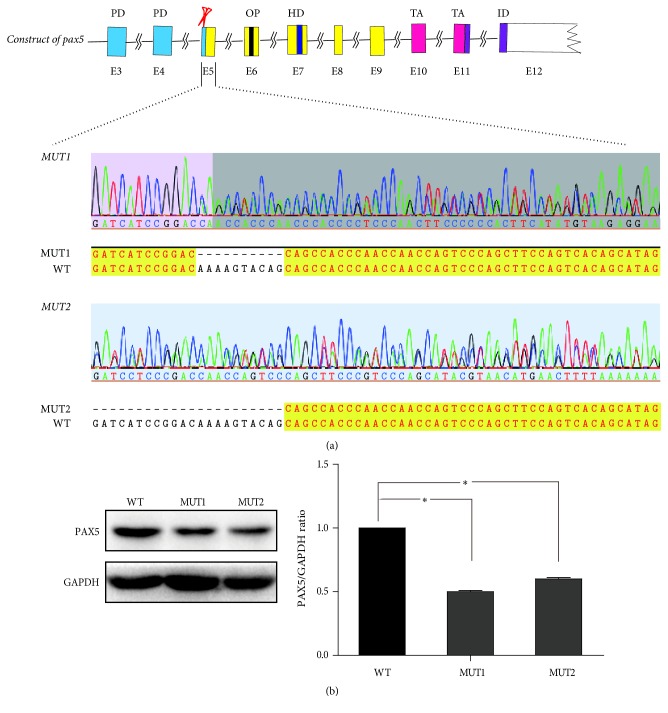
Mutation type of pax5 and identification. (a) The construction of pax5 and mutation type of pax5. E5 was the target of gene editing operation, and sequencing analyses identified knocked out regions of E5. (b) Protein level of pax5 tested by western blot. Protein level of pax5 in MUT1 and MUT2 was less than 50% of that in WT. E: exon of pax5; PD: paired box domain; OP: octapeptide domain; HD: homeodomain; TA: transactivation domain; ID: inhibitory domain; WT: wild-type clone; MUT: mutation clone. *∗* means *p* < 0.05.

Targeted genome editing tools such as CRISPR-Cas9 system have been widely used to modify genes in model systems including animal and human cells [22]. We designed a gRNA that directed exon5 of PAX5, which was shared by all different transcripts, and we inverted it into the cas9 and GFP expressing vector. After the editing and subsequent screening process, we randomly picked out the clones and screened out mutated clones by DNA sequencing. Sequencing results showed that mutation clones had haploid deletion mutation in exon5 of PAX5 by comparison of DNA sequences of mutation clone with that of wild-type clone (Figure 1(a)). Haploid deletion mutation resulted in haploid termination of PAX5 mRNA transcription. Western blot showed that PAX5 protein level in mutation clone was significantly lower than that in wild-type clone (*p* < 0.05) (Figure 1(b)).

### 3.2. PAX5 Haploinsufficiency Induced the Differentially Expressed Genes (DEGs)

After analyzing the microarray data in PAX5+/− Raji cells and their mother wild-type cells, we screened out a total of 213 DEGs in PAX5+/− Raji cells (MUT1) compared to mother wild-type cells, including 82 downregulated genes and 131 upregulated genes. A total of 199 DEGs were screened out in PAX5+/− Raji cells (MUT2) compared to mother wild-type cells, including 85 downregulated genes and 114 upregulated genes. There were 135 common target genes, including 47 downregulated genes and 88 upregulated genes.

### 3.3. The Motif Analysis of PAX5 and Identification of PAX5 Target Genes

**Figure 2:**
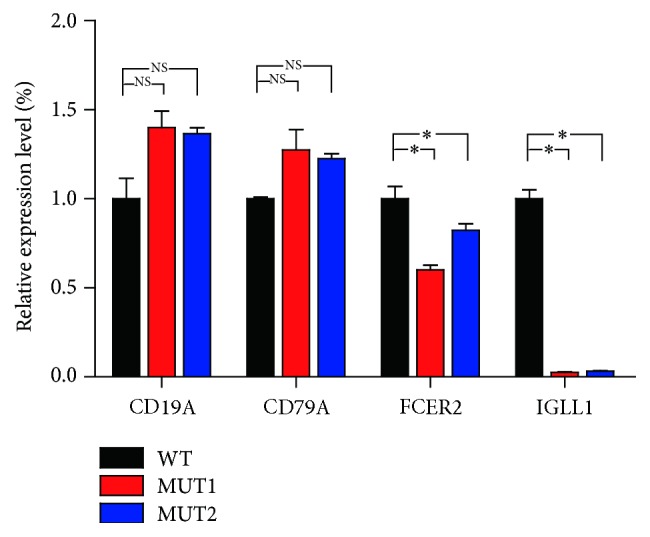
Target genes tested by quantitative RT-PCR. The expression of CD19 and CD79A was not changed. The expression of IGLL1 and FCER2 was decreased. ^NS^*p* > 0.05 versus WT, ^*∗*^*p* < 0.01 versus WT.

Based on motif analysis of PAX5, we searched out 28 PAX5 target genes, that is, BAX, BCL2, BLK, CCND1, CD19, D79A, CDKN1A, FCER2, FHL2, LEF1, MET, MMP1, MYCNA, PRDM1, PRKCE, RAG2, RB1, TIMP1, TP53, XBP1, EGR1, ESR1, ELK1, ETS1, KCNH4, VPREB1, IGLL1, and KCNH8. Two differentially expressed genes (IGLL1 and FCER2) were identified as targets of PAX5. The expression of IGLL1 and FCER2 was decreased in PAX5+/− Raji cells. CD19 and CD79A are recognized target genes of PAX5, but we found that the expression of CD19 and CD79A was not changed from the microarray data. In order to verify the results, we detected mRNA level of CD19, CD79A, IGLL1, and FCER2 using quantitative PCR. Results showed that the expression of IGLL1 and FCER2 was decreased and the expression of CD19 and CD79A was not changed (Figure 2).

### 3.4. Construction of PPI Network

**Figure 3:**
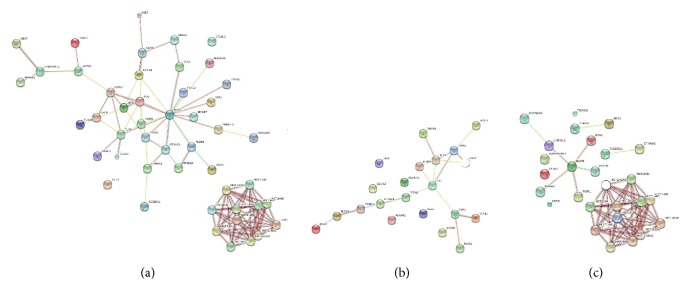
The differentially expressed genes were used to search the STRING database to predict their protein-protein interactions in pax5 mutation cells. In the network shown, the nodes are the proteins and the lines represent the predicted functional associations. The number of lines indicates the strength of the predicted functional interactions of the proteins. (a) Network of differentially expressed genes (DEGs). (b) The downregulated DEG PPI subnetwork. (c) The upregulated DEG PPI subnetwork.

There were 135 common target genes, which were critical to explore the potential roles of PAX5 haploinsufficiency. The analysis of PPI networks found that there were 49 genes which were related to each other. The total DEGs PPI network contained 39 nodes and 157 edges (interactions), including 21 downregulated DEGs and 28 upregulated DEGs (Figure 3(a)). The downregulated DEG PPI subnetwork contained 21 nodes and 17 edges (Figure 3(b)). The upregulated DEG PPI subnetwork contained 28 nodes and 61 edges (Figure 3(c)). These three networks indicated that PAX5 haploinsufficiency greatly disturbed the PPI network in Raji as DEGs interactions change the biological consequences. We found all histones constructed one unattached network, and there were several important node genes such as EGFR, FOS, HSPA5, TLR4, and MMP9 (Figure 3).

### 3.5. Functional Annotation Clustering of DEGs in Network Using the DAVID

Using the DAVID, we did functional annotation cluster analysis for 49 interconnected genes. DAVID functional clustering of 49 genes returned 18 clusters, and there were 5 clusters with enrichment scores > 1 (*p* < 0.05) (Table 1). These significant clusters reflected the notion that the dataset was representative of basic biological processes. PAX5 haploinsufficiency had the greatest influence on clusters involved in gene transcription, inflammatory and immune response, and cancer pathways. There were 17 genes involved in gene transcription, including 12 histone cluster genes. Six genes of DEGs in network participated in inflammatory and immune response. Seven genes of DEGs in network participated in pathways in cancer. Other clusters included cell adhesion, protein phosphatase, and tight junction.

## 4. Discussion 

PAX5 haploinsufficiency occurred frequently in lymphocytic neoplasm and plays an important role in lymphocytic tumorigenesis. Our report described the characteristics of changed gene expression after PAX5 was haploidentically knocked out in Raji cells.

We obtained two PAX5+/− Raji cell clones by targeted genome editing tools. Western blot showed that PAX5 protein level in PAX5+/− Raji clones was significantly lower than that in wild-type Raji clone. Based on motif analysis of PAX5, two differentially expressed genes (IGLL1 and FCER2) were identified as targets of PAX5. The expression of IGLL1 and FCER2 was decreased in PAX5+/− Raji cells. CD19 and CD79A, which are recognized target genes of PAX5, were not changed from the microarray data. Quantitative PCR confirmed microarray data. Expression of PAX5 in pro-B cells activates the genes such as CD19 and CD79A which were required for differentiation toward B-cells. Ex vivo, PAX5−/− pro-B cells are not restricted to the B-lymphoid lineage [23]. Clare Pridans performed a global gene expression screen of wild-type (PAX5+/+) and PAX5-deletion (PAX5−/−) pro-B cells in an attempt to identify the crucial PAX5 targets. The expression of CD19 and CD79A was decreased in PAX5−/− pro-B cells compared to that in PAX5+/+ pro-B cells [16], but there was no change in expression of IGLL1 and FCER2. Our report showed opposite results. The expression of CD19 and CD79A was not significantly decreased in PAX5+/− Raji cells compared to that in PAX5+/+ Raji cells, and the expression of IGLL1 and FCER2 was significantly decreased in PAX5+/− Raji cells. So, we believe that the genetic variation characteristics caused by PAX5 haploid deletion were different from that caused by PAX5 diploid deletion. PAX5 haploinsufficiency has been reported as being frequently altered in adult B-ALL [7]. Analysis of genetic variation characteristics caused by PAX5 haploid deletion was very important for clarifying the role of PAX5 haploid deletion in tumorigenesis. We analyzed mRNA expression profile from two PAX5+/− Raji cell lines; only 135 common target genes were selected. The analysis of PPI Networks found that there were only 49 genes which were related to each other. These genes were divided into two networks: one network was interlocking histone genes clusters and another network consisted of 39 genes, which included several important node genes such as EGFR, FOS, HSPA5, TLR4, and MMP9 (Figure 3).

DAVID functional clustering of 49 genes returned 18 clusters, and there were 5 clusters with enrichment scores 1. These significant clusters reflected that the dataset was representative of basic biological processes. These processes included gene transcription, inflammatory and immune response, tumor pathway, cell adhesion, and tight junction.

The processes where transcription factors regulate target genes are complicated, and histones play important roles during these processes. Histone gene clusters are heterogeneously organized and contain 1 or more copies of the 5 histone subtypes, that is, core (H2A, H2B, H3, H4, and variants thereof) and linker (H1) histone genes [24]. Histones determine target genes expression, and histone mRNAs are closely regulated during the cell cycle, permitting the synthesis of histone proteins to occur coordinately with the replication of DNA. Our results showed that 12 histone genes were all upregulated; these genes were all related to gene transcription. In tumors, upregulation of histone mRNA indicates proliferative activity of tumor cells. Many studies have demonstrated that histone mRNA accumulates in tumors [25–28] and histones control target genes expression. These histones may take part in the process where PAX5 haploinsufficiency causes gene expression changes. In our identified histones, previous literatures showed that HIST1H2BF, HIST1H3B, HIST1H4C, and HIST1H3D took part in tumorigenesis [28–31]. Many previous researches showed that other genes related to gene transcription were involved in tumorigenesis and prognosis [32–34]. Chromatin modifications implicated in transcriptional regulation are thought to be the result of a code on the histone proteins [35]. Abnormal expression of histone genes caused genomic instability, which enabled cells to acquire genetic alterations that promoted oncogenesis [36]. We guessed that the role of PAX5 haploinsufficiency in tumorigenesis might be partially achieved by PAX5 haploinsufficiency inducing genomic instability, although there was no report about PAX5 haploinsufficiency inducing genomic instability.

PAX5 is a member of the PAX family of developmental transcription factors with an important role in B-cell development. PAX5 activates the chromatin of key genes involved in B-cell signaling, adhesion, migration, and immune function [37]. The genes involved in inflammatory and immune response included TICAM2, CCL4L2, TLR4, FOS, AKT3, and PTPN6. These genes took part in tumorigenesis [38–42]. TLR4, Fos, and AKT3 were primary node genes. PAX5 haploinsufficiency induced decreased expression of TLR4 and Fos and induced increased expression of AKT3. TLRs play important roles in regulating innate immune responses. TLR4 controls the host defense by sensing an exotic pathogen. TLR4 is often overexpressed in malignant and tumor-infiltrating immune cells, and the application of TLR4 ligands in cancer therapies is desirable for enhancement of antitumor immunity [38, 39]. PAX5 haploinsufficiency induced decreased expression of TLR4, and the downregulated TLR4 participated in immune tolerance [43, 44]. Fos was involved in inducing a large number of cytokine genes and other genes that were central to the productive immune response. The absence of Fos induced immune tolerance [45, 46]. AKT3 is involved in a variety of biological processes. Highly expressed AKT3 participated not only in tumorigenesis [47] but also in autoimmune encephalomyelitis and graft-versus-host response [40, 48]. No study was reported about PAX5 haploinsufficiency involved in immune tolerance, but we had evidence to assume that PAX5 haploinsufficiency may result in tumor immune tolerance.

Cancer pathways participated in cancer pathophysiological processes. PAX5 haploinsufficiency caused the abnormal expression of genes involved in cancer pathways. Increased genes included EGFR, FGF12, AKT3, and CTNNA3 (Figure 3(c), Table 1). EGFR is a transmembrane tyrosine kinase receptor involved in the regulation of cellular multiplication, survival, and differentiation. EGFR is identified as cellular protooncogene and is overexpressed in a variety of human cancers [49]. EGFR inhibitors were used to treat not only non-small-cell lung cancer with EGFR mutation, but also cancer with overexpression of EGFR [50]. FGF12, as one proapoptotic gene, suppresses radiation-induced apoptosis through p38*α* [51, 52]. FGF12 was identified as a new potential marker for prostate tumors [53, 54]. AKT, a major downstream mediator of PI3K pathway, was shown to regulate cancer progression. Three highly homologous AKT isoforms (i.e., AKT1, AKT2, and AKT3) may play different roles. Increased AKT3 expression not only promoted prostate cancer proliferation [55], but also conferred resistance to AKT inhibitor in breast cancer [56] and PLK inhibitors in human colorectal cancer [57]. Inhibiting AKT3 and PI3KCA enhanced chemotherapy sensitivity in glioblastoma multiforme cells [47].

## 5. Conclusion

In conclusion, our study here shows that changes in the gene transcription, inflammatory and immune response, and cancer pathways were identified as three important pathways associated with PAX5 haploinsufficiency. These changes hinted that the mechanism of PAX5 haploinsufficiency promoting tumorigenesis may be related to genomic instability, immune tolerance, and tumor pathways.

## Figures and Tables

**Table 1:** Functional annotation clustering in the DAVID disease/cancer database.

Cluster	Count	Score	Genes
Gene transcription	17	2.31	HIST2H3A, HIST1H2BC, HIST1H2BF, HIST1H2AD, HIST1H3B, HIST1H4E, HIST1H3D, HIST1H4C, HIST1H2AM, HIST1H4I, HIST1H2AL, HIST2H3C, EGFR, FOS, CALR, EYA1, MAGI1

Inflammatory and immune response	6	1.42	TICAM2, CCL4L2, TLR4, FOS, AKT3, PTPN6

Pathways in cancer	7	1.39	EGFR, CCNE1, FOS, MMP9, FGF12, AKT3, CTNNA3

Cell adhesion	7	1.38	EGFR, CNTNAP4, MAGI1, CTNND1, CCL4L2, CTNNA3, PTPN6

Protein phosphatase	7	1.34	EGFR, PTPN6, EYA1, PTPRG, ROR1, AKT3, CDK3

Tight junction	4	1.03	EPB41L3, MAGI1, AKT3, CTNNA3
